# A Wavelet Transform-Based Neural Network Denoising Algorithm for Mobile Phonocardiography

**DOI:** 10.3390/s19040957

**Published:** 2019-02-24

**Authors:** Dawid Gradolewski, Giovanni Magenes, Sven Johansson, Wlodek J. Kulesza

**Affiliations:** 1 Blekinge Institute of Technology, Institute of Applied Signal Processing, 371 79 Karlskrona, Sweden; sven.johansson@bth.se (S.J.); wlodek.kulesza@bth.se (W.J.K.); 2 Dipartimento di Ingegneria Industriale e dell’Informazione, University of Pavia, 27100 Pavia, Italy; giovanni.magenes@unipv.it

**Keywords:** adaptive filters, auscultation techniques, auto-diagnostic system, cardiovascular pathologies, Inverse Wavelet Transform (IWT), noise cancellation, signal denoising, Time Delay Neural Networks (TDNN)

## Abstract

Cardiovascular pathologies cause 23.5% of human deaths, worldwide. An auto-diagnostic system monitoring heart activity, which can identify the early symptoms of cardiac illnesses, might reduce the death rate caused by these problems. Phonocardiography (PCG) is one of the possible techniques able to detect heart problems. Nevertheless, acoustic signal enhancement is required since it is exposed to various disturbances coming from different sources. The most common denoising enhancement is based on the Wavelet Transform (WT). However, the WT is highly susceptible to variations in the noise frequency distribution. This paper proposes a new adaptive denoising algorithm, which combines WT and Time Delay Neural Networks (TDNN). The acquired signal is decomposed by means of the WT using the coif five-wavelet basis at the tenth decomposition level and then provided as input to the TDNN. Besides the advantage of adaptive thresholding, the reason for using TDNNs is their capacity of estimating the Inverse Wavelet Transform (IWT). The best parameters of the TDNN were found for a NN consisting of 25 neurons in the first and 15 in the second layer and the delay block of 12 samples. The method was evaluated on several pathological heart sounds and on signals recorded in a noisy environment. The performance of the developed system with respect to other wavelet-based denoising approaches was validated by the online questionnaire.

## 1. Introduction

Auscultation techniques, generally performed using a stethoscope, allow a quick examination of the condition of circulatory, respiratory or gastrointestinal systems by listening to breath, heart or bowel sounds, respectively. These simple methods have no risk or side effects and allow a fast evaluation of the respective systems’ conditions. Because of their features, general practitioners use auscultation methods at an early stage of diagnosis. However, using a stethoscope, which only acquires the sound level, still requires much practice and experience. In the phonocardiography (PCG)-based stethoscope, it is most useful to listen for sounds and murmurs of the heart resulting from the vibrations created by the closure of heart valves and turbulent blood flow through the vessel [1].

According to the World Health Organization (WHO), failures of the cardiovascular system cause 23% of deaths all over the world [2]. Early recognition of pathological changes could save many lives. Therefore, the development of a reliable auto-diagnostic system, which could be applied to both home healthcare and in a clinical environment, is in great need. Phonocardiography, due to its simplicity and non-invasiveness, is a possible solution and could facilitate monitoring of both hospitalized people and those whose health conditions can be checked at home.

**Figure 1:**
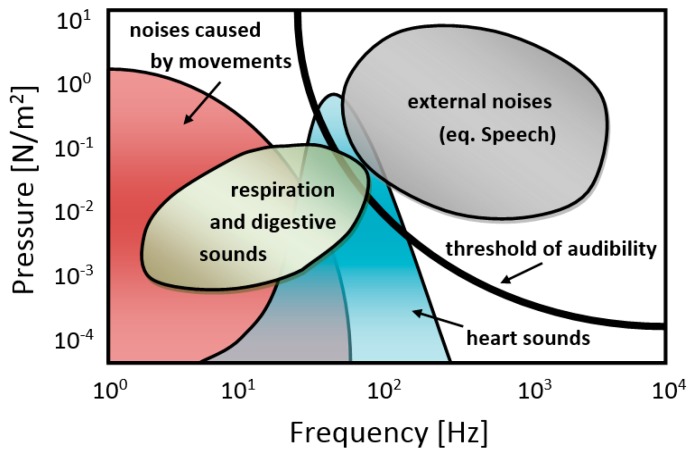
Spectral intensity map of PCG and related disturbance signals (source [3,6]).

The main problem of such a system are the interferences that occur during acquisition of the PCG signal (Figure 1) [3,4,5,6]. There are interferences of external origin derived from surroundings, such as speech or external sounds, and of internal origin mainly caused by respiratory and digestive sounds, as well as those induced by patients’ movements [3,4]. Occasionally, some other disturbances may occur from sensor rubbing, swallowing, muscle movements, coughing, etc. [5].

## 2. Survey of Related Works

Several attempts were made to develop reliable denoising algorithms for PCG signals. Adaptive algorithms represent a possible approach [6,7] relatively effective to remove human’s internal sounds like respiration [8] or digestive sounds [9]. However, these methods require additional sensors placed on the patients’ body, making the test inconvenient and difficult to implement as a mobile or wearable system capable of working remotely in patients’ homes [10,11,12]. Furthermore, the problem of external origin disturbances, like movements [3] or speech [4], requires the implementation of suitable filters.

Another category of PCG denoising algorithms refers to blind source separation techniques grounded on some quasi-periodic properties of heart sounds [13,14]. Among these solutions, the model-based Bayesian denoising framework, developed by Almasi et al. [15], and the single channel method proposed by Jimenez-Gonzalez and James [16,17] show promising results. The similarities of spectral features were also used for detection of ambient, vocal and physiological disturbances of PCG signals [18].

Nowadays, the common approaches are based on the wavelet thresholding algorithms [4,19,20,21,22,23,24], which are also widely applied for a denoising processes of other bio-signals such as ECG [25,26] or EMG [27,28]. The Wavelet Transform (WT), due to its high resolution both in time and frequency domains, has also been successfully used for PCG signal processing [29,30,31] and feature extraction [32,33].

Naseri et al. applied the WT to binary quality assessment system [20] and noise/spike detection in PCG signals [21]. The authors of [19] adopted the WT-based denoising technique in PCG signal filtration. They found that the *rigsure* thresholding method and the non-rescaling *sln* function are suitable to remove white noise from heart sound signals. Liu et al. showed good results with the *minimaxi* thresholding algorithm [23]. In [4], the authors reported that the noise recorded by a mobile PCG acquisition device in a noisy environment had a distribution similar to pink noise, and the *minimaxi* thresholding method and *mln* function, rescaled using a noise level dependent estimation, are suitable to remove this environmental noise. Cherif et al. observed that the Discrete Wavelet Transform (DWT) more efficiently removes murmurs and clicks than the Packet Wavelet Transform (PWT) [29]. Overall, many wavelet-based denoising approaches using thresholding algorithms have been proposed to improve the PCG signal quality [4,19,23,24,29].

It has been observed that the optimal parameters of the wavelet denoising algorithm for a PCG signal [4,19,20,21,22,23,24] depend on the initial simulation conditions [21]. The use of an adaptable threshold value might be suitable for systems working in variable surrounding environments, where the sources of noise change instantaneously [5,21]. Consequently, several recent studies approach the problem of automatic determination of the threshold value [34,35]. An adaptive overlapping-group sparse denoising heart sound signal algorithm proposed by Deng and Han outperforms the conventional wavelet methods in lower noise level [36]. An adaptive threshold estimation method for wavelet based denoising reported by Jain and Tiwari estimates the threshold value on the basis of domain knowledge about the heart sound signal [37]. The algorithm efficiently distinguishing heart murmur from dataset using the wavelet transform and combination of artificial neural network was developed by Eslamizadeh and Barati [38].

## 3. Problem Statement and Main Contribution

The review of related works shows that the existing auto–diagnostic methods require enhancement of the PCG denoising system. Most of the research focuses on removing white noise contamination. However, recent studies demonstrate that the sources of noise on the PCG signal vary widely [4], affecting the power distribution of the disturbance signals. Therefore, there is a need for a denoising system adaptable to various noises, regardless of their origin, power or distribution. Furthermore, the reported studies do not consider that some of the useful heart sounds such as snaps, rumbles or murmurs are similar to noise and can be unduly affected by a filtration process changing the signal morphology.

The main objective of the paper is to design of a phonocardiography denoising algorithm adaptable to the changing surrounding interferences without compromising its complexity and usability.

The proposed denoising system combines two techniques: WT and the Time Delayed Neural Network (TDNN), where the WT decomposes the PCG signal to provide its valuable frequency content to the TDNN. Whereas, the TDNN, besides filtering the wavelet coefficients below the adaptively adjusted threshold, estimates the Inverse Wavelet Transform (IWT) from the wavelet coefficients exclusively associated with the desired heart sound.

The main contribution of the paper is the modelling and implementation of the new adaptive denoising algorithm aimed at the self-adjustment to the changeable surrounding environment. The proposed wavelet-based Neural Network (NN) denoising method was modelled and then implemented in Matlab. The design of the TDNN is based on two optimisation parameters, Signal to Noise Ratio (SNR) and fit coefficient. The solution evaluation has been performed on several pathological heart sounds and signals recorded by mobile devices in a noisy environment. The performance of the developed system, with respect to other wavelet-based denoising approaches [4,19], was validated by an online questionnaire. Moreover, the proposed solution was verified using the heart sound classifier.

## 4. System Architecture

The proposed system combines two methods: Wavelet Packet Decomposition (WPD), known also as Wavelet Packets or Subband Tree, and the TDNN. The used decomposition technique applies a series of low-pass and high-pass filters. The used two-channel sub-band coder was developed by Mallat [19,39,40,41].

In [4,19], it was shown that WPD provides a sparse representation of PCG signals, wherein the coefficients of small value represent the noise, while the main signal features are included in a few large-magnitude wavelet coefficients [19]. Therefore, the proposed denoising algorithm aims at removing the irrelevant wavelet coefficients, exclusively attributed to the noise, in order to reconstruct of the originally desired signal through the remaining informative coefficients [41]. During the training process, based on noise input and clean output data, the NN learns which part of signal is relevant, and which is associated with noise and should be removed. However, it is crucial to find the relevant threshold value for preserving the only the desired coefficients [21]. To fulfil this request, instead of finding a constant threshold value, we apply a NN, which reconstructs the desired heart sound signal based on the recorded signal and corresponding wavelet coefficients. The purpose of the NN is to preserve only these wavelet coefficients, which contain heart sound features and then to estimate the IWT. We used the TDNNs due to the time series data [42] and ability to recognize the series features independent of time-shift [43,44].

**Figure 2:**
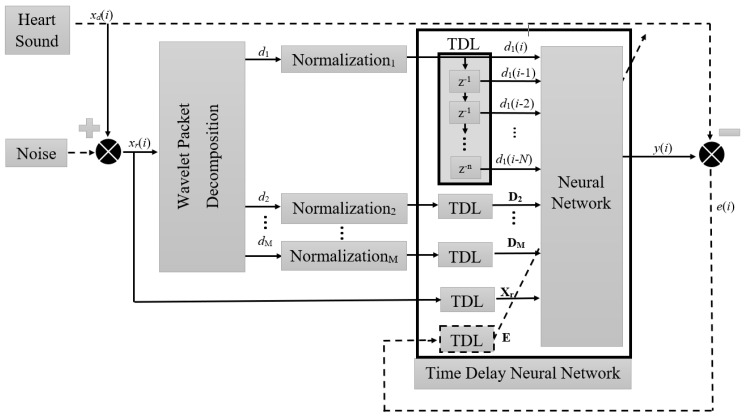
The block diagram of the proposed system, the dashed lines represent the training scheme of NN.

The block diagram of the proposed system is presented in Figure 2. The core of the system is the TDNN placed after the WPD. Previous studies [4,19] show that, when dealing with PCG signals affected by noise of varying distribution, the best parameters for WPD can be obtained by means of *Coif* 5 wavelet basis using *M* = 10 decomposition levels [29]. Therefore, these parameters are applied in our solution. The wavelet detail coefficients (*d*_1_ − *d_M_*) of the decomposed signal are resampled to get TDNN input data of the same length. The PWD coefficients are up-sampled by placing a sample value of the previous coefficients, which duplicates the length of the series. The Tapped Delay Line (TDL) of the NN delays the signal by *n* samples, therefore, each created vector is made up of the current time wavelet coefficients *d_k_*(*i*) and *N* coefficients of the delayed samples. The NN applies *N*-samples of each normalized wavelet coefficient vectors (**D**_1_ − **D**_M_) and real signal **X_r_** to estimate the desired signal *x_d_*(*i*), where ***D****_j_* = [*d_j_*(*z*) *d_j_*(*z* − 1) … *d_j_*(*z* − *N*)] and **X_r_** = [*x_r_*(*z*) *x_r_* (*z* − 1) … *x_r_*(*z* − *n*)] and *j* is a decomposition level. Each of the NN inputs collects data for time windows with different lengths.

The logical layout for the NN training process of the applied denoising system is also depicted in Figure 2, where the dashed lines represent the training scheme of the NN. The desired heart sound signal *x_d_*(*i*) is compared with the NN response *y*(*i*), and thus the output error *e*(*i*) is used to train the TDNN using a backpropagation algorithm [44].

## 5. Design of the TDNN

To determine the best configuration and its parameters of the TDNN, the system performance is evaluated for various doses of white and pink noise. The noise was added to desired heart sound signals with gradually increasing power from 1 dBm to 15 dBm, with an incremental step of 1 dBm. As a quality measure of the proposed system, the SNR of the desired signal to the denoised signal was used. Moreover, to ensure that during filtration important information is not lost, an adopted *fit* coefficient was proposed:

(1) $$fit=100\times \left(1-\frac{\sum_{j=1}^{L}{\left[y\left(j\right)-x_{d}\left(j\right)\right]}^{2}}{\sum_{j=1}^{L}{\left[x_{d}\left(j\right)-\frac{1}{L}\sum_{j=1}^{L}x_{d}\right]}^{2}}\right),$$

where *L* is the number of the signal samples, *x_d_* is the desired signal and *y* is the denoised signal.

The proposed *fit* is the normalized complement to one of the determination coefficients [45], commonly applied to evaluate ECG [25] and PCG [4,6] denoising systems. The greater *fit* value denotes better matching between the desired and denoised signals.

### 5.1. Bases of Heuristic Design Optimisation

For system heuristic design optimisation, we used records from five online databases: Michigan [46], eGeneral Medical Inc. [47], 3M Litttmann [48], University of Washington [49] and Thinklabs [50]. Each dataset consists of 10 different sets of records including physiological heart sounds (S1 - S4), as well as sounds that indicate the occurrence of a cardiovascular pathology: Normal Split S1 (NS S1), Normal Split S2 (NS S2), Early Systolic Murmur (ESM), Late Systolic Murmur (LSM), Ejection Click (EC), Opening Snap (OS), Pansystolic Murmur (PM), and Diastolic Rumble (DR). The overview of databases’ dataset is presented in Table 1.

The sounds were recorded at different sampling frequencies, varying from 8 kHz to 11 kHz. For standardization purpose and to reduce the computational complexity the records of all databases were down-sampled to the common frequency of 2000 Hz, without losing the signal quality. The database sets were divided into two equal sets, one for training and one for testing. The training and testing sets are composed of randomly selected samples from each sound and each online available database. Both sets contained the same number of recordings of normal (S1, S2, S3, S3) and pathological (EC, NS S1, NS S2, LMS, HM, ESM, OS, DR) heart sounds. In training phase, to the training recordings, the uncorrelated Gaussian white and pink noises were added respectively, each of 5 dBm, 10 dBm, and 15 dBm levels. The proposed system was evaluated using the test recording set contaminated by the gradually increasing white and pink noises from 1 dBm to 15 dBm, with a step of 1 dBm. The average *fit* coefficient and the output SNR obtained for each test sound signal were analysed at each noise level. 

### 5.2. Heuristic Rstimation of the TDNN Parameters

The WPD parameters of 10th level and *Coif* 5 wavelet basis have already been determined in [4,19,23]. Therefore, we only needed found TDNN parameters: *n*—the size of the tapped delay line, and the number of neurons used in hidden layers of the Artificial Neural Network (ANN).

The Levenberg-Marquardt backpropagation training algorithm [44] (*trainlm* in Matlab toolbox) was chosen for the training process, and the evaluation was carried out by means of the Mean Squared Error (*MSE*). To find the global minimum of the gradient function for each simulation, 500 epochs were empirically chosen.

**Figure 3:**
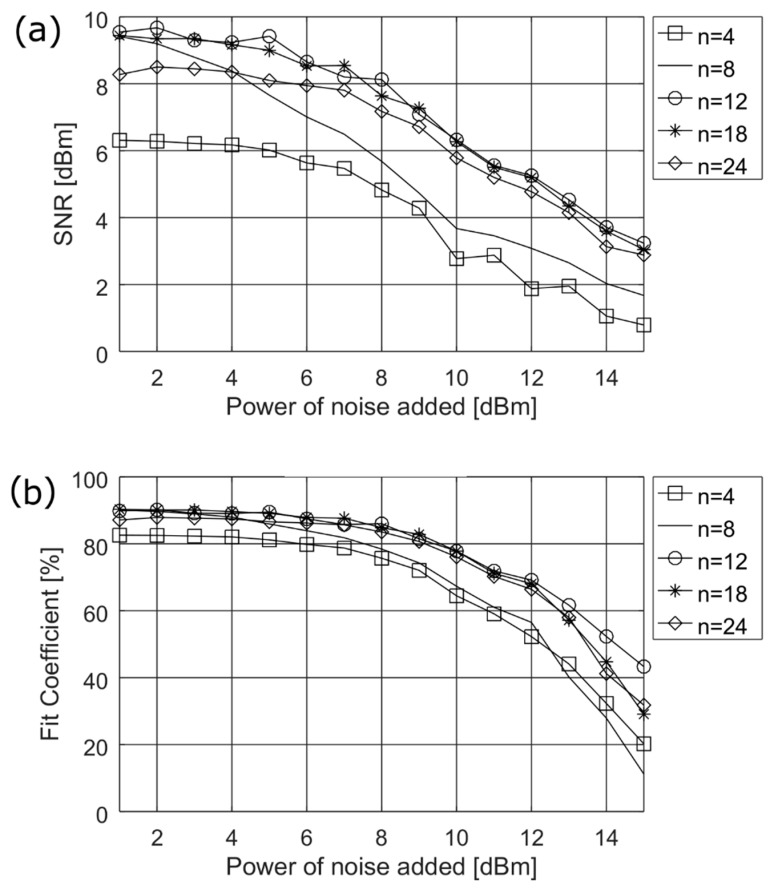
Simulation results obtained during determination of the optimum length for tapped delay line—*n*, (**a**) of SNR and (**b**) of fit coefficient.

Figure 3 presents the simulation results used to find the optimum *n* length of tapped delay line for the five representative values of TDL block (*n* = 4, 8, 12, 18, and 24). The upper limit of the delay *n* = 24 should not be exceeded due to the algorithm computational complexity of the training process and because of the implementation requirements of the TDNN for *real time* applications, e.g., on mobile devices. The simulations were performed on a medium size ANN containing 15 neurons in the hidden layer. From Figure 3, it can be noticed that the best values of both SNR and *fit* coefficient are obtained for *n* = 12 delays, which is especially distinguishable for *fit* coefficient. However, the values of *n* = 24 and *n* = 18 do not differ much especially in respect to SNR.

The final design step was to find the suitable structure of the NN. In Table 2, six representative medium size networks used in simulation are defined. ANN1, ANN2, ANN3 and ANN4 were built with a single hidden layer of 10, 15, 20, and 25 neurons, respectively. ANN5 and ANN6 consisted of two hidden layers with 25 neurons in the first layer and 15 neurons and 20 neurons in the second layer, respectively. Each NN consisted of one output layer.

**Figure 4:**
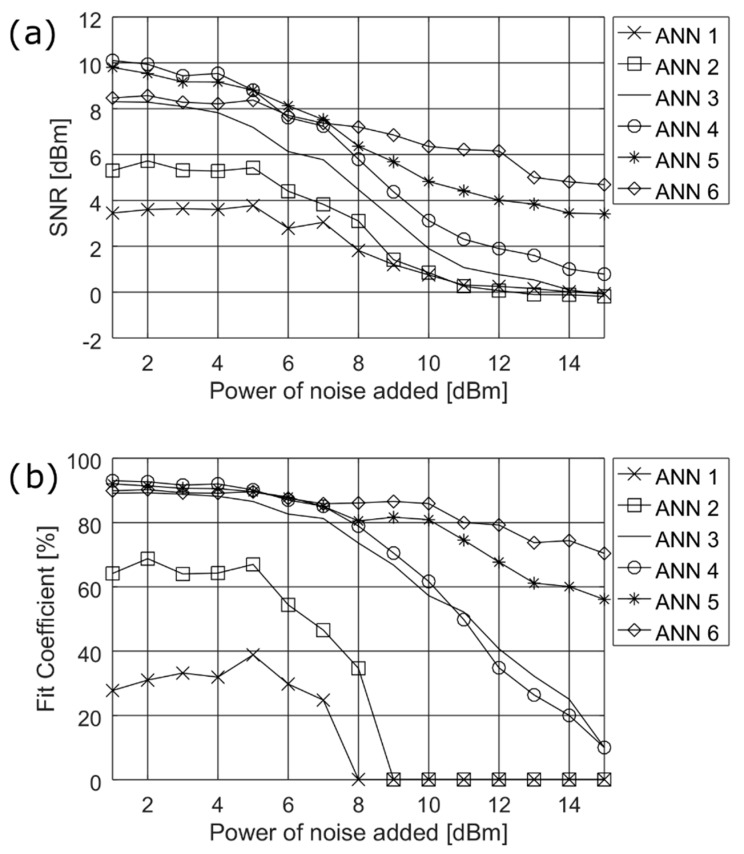
Simulation results for finding the most suitable structure of the NN, (**a**) of SNR and (**b**) of fit coefficient.

The simulation results of SNR and fit coefficient presented in Figure 4 show the parameters’ worst performance for the single layer neural networks (ANN1–ANN4). ANN5 and ANN6 show similar good performance. However, ANN6 with 25 neurons in first and 20 neurons in the second layer, performs slightly better at higher noise levels, and, therefore, it can be recommended for our solution.

Finally, the designed wavelet-based NN filter consists of 25 neurons in in the first and 20 neurons in second layer and has the delay block of 12 samples (*n* = 12). The wavelet decomposition parameters, based on previous studies [4,19] are determined as *Coif* 5 wavelet basis at the 10th decomposition level.

## 6. System Evaluation

In the evaluation process, we compared the performance of the proposed system with the wavelet denoising filters (WDF) based on a constant threshold value optimized for pink and white noise. The simulations were performed on:

- WDF optimised for white noise and contaminated by white noise [19] (WD white _w_),
- WDF optimised for white noise and contaminated by pink noise [19] (WD white _p_),
- WDF optimised for pink noise and contaminated by white noise [4] (WD pink _w_),
- WDF optimised for pink noise and contaminated by pink noise [4] (WD pink _p_),
- Wavelet Transform Time Delay Neural network contaminated by white noise (WT-TDNN _w_),
- Wavelet Transform Time Delay Neural network contaminated by pink noise (WT-TDNN _p_).

**Figure 5:**
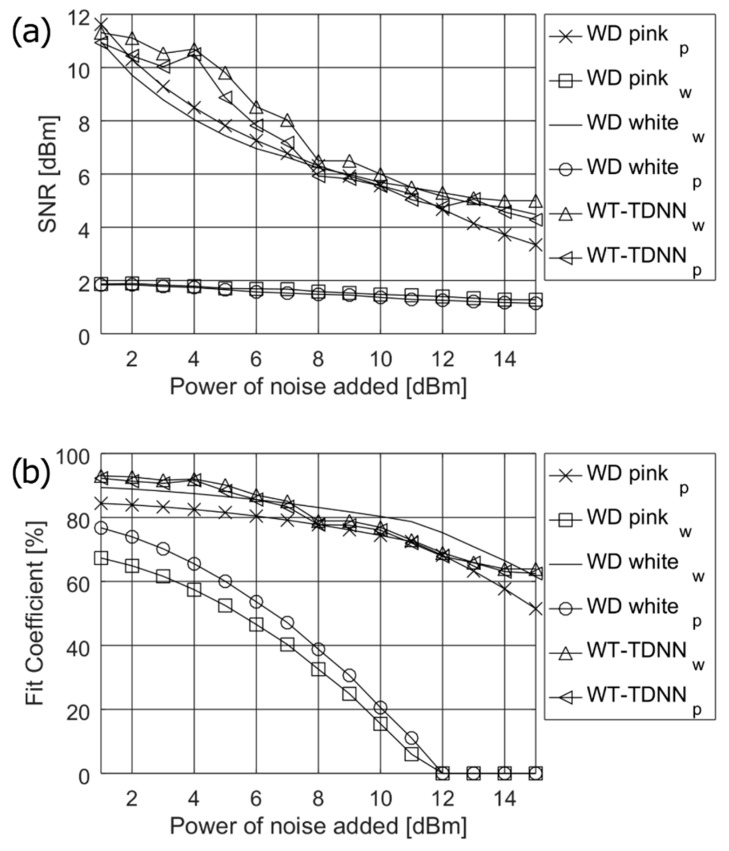
The performance comparison of the developed system with NN and wavelet denoising algorithms, (**a**) of SNR and (**b**) of fit coefficient.

The power of additive noise was gradually increased from 1 dBm to 15 dBm, with a step of 1 dBm, separately for both white and pink noise contaminations. The results are presented in Figure 5 and show that the wavelet denoising filters based on a constant threshold value [4,19] are highly susceptible to changes in the noise distribution and optimisation target. In the case of contamination by the same noise distribution as used in the optimization process, their denoising capabilities are similar to those of the proposed systems in terms of SNR and fit coefficient. However, in the case of contamination by a different noise distribution than that used in the optimization process they perform poorly (SNR <2.5). The best results independent of distribution of noise contaminants were obtained by using the proposed wavelet-based NN denoising algorithm.

## 7. System Validation

**Figure 6:**
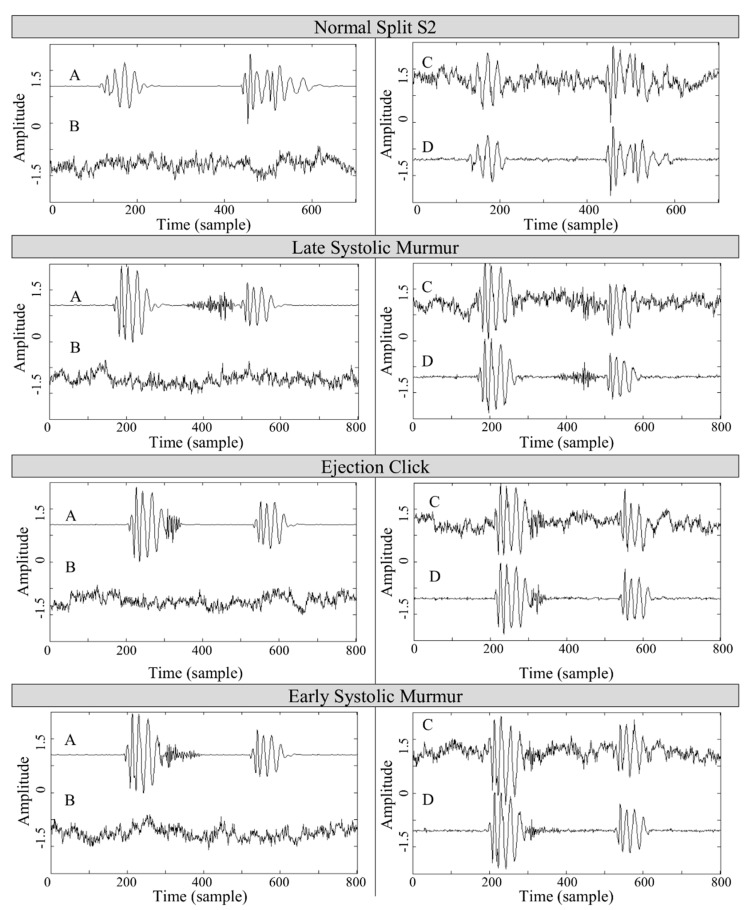
The final results of the filtration process. A—desired signal, B—the pink noise of SNR = 10 dBm, C—noised signal, and D—denoised signal.

**Figure A1:**
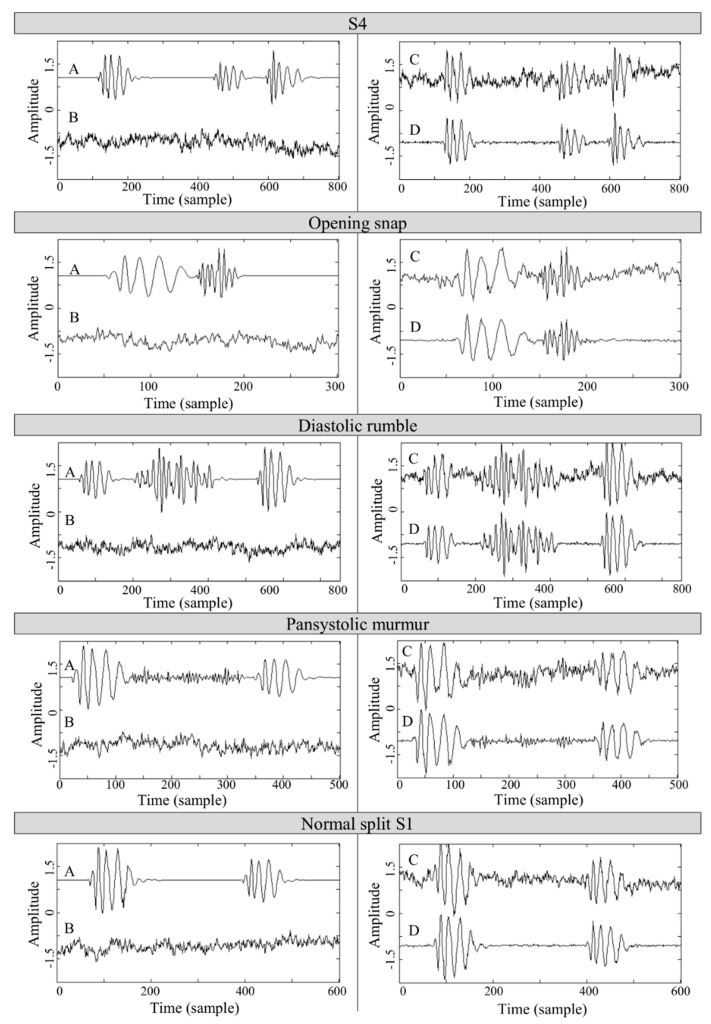
The final results of the filtration process. A—desired signal, B—the pink noise of SNR = 10 dBm, C—noised signal, and D—denoised signal.

Some examples relevant to heart sound signals filtered by the proposed system are presented in Figure 6 showing one example for each snap, rumble, murmur, split, and a physiological heart sound. Supplementary simulations are enclosed in the Appendix A in Figure A1. In order to allow an easy interpretation of the denoising accuracy of the developed system, the results depicted in Figure 6 and Figure A1 are organized as follows: the left frame contains the desired signal *A*, the 10 dBm pink noise *B* added to the desired signal, the right frame contains the resultant noised signal *C*, and the signal after denoising *D*. It can be observed that after filtration, a residual low amplitude high frequency noise is still present the denoised signal. Nevertheless, it can also be seen that after the filtration process the morphology of the denoised and original signals remains mostly unchanged. Although some components of the original heart signal are removed, mainly murmurs, the original denoised signal is recognizable and its tones distinguishable.

**Figure 7:**
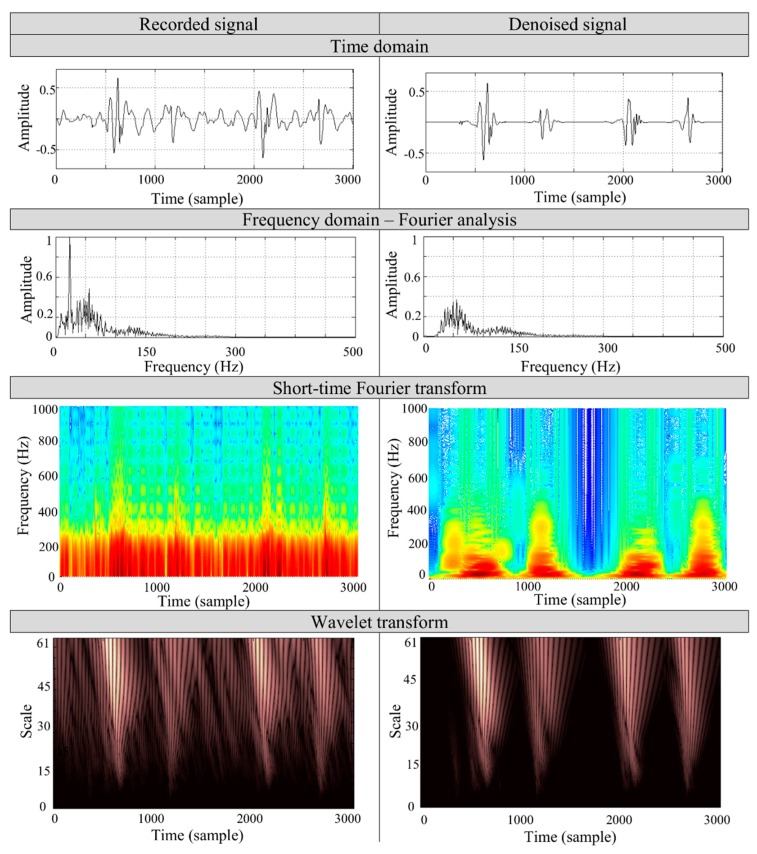
The results of the denoising of real PCG signal containing of two heart cycles recorded in a noisy environment by mobile devices with corresponding spectrum analysis.

Figure 7 presents the case study of the denoising process on a PCG signal of a 27 year old woman, recorded by a mobile device [4] in a noisy environment. During the measurement, the noise sources of the TV set and computer of noise levels about 65 dB and 60 dB, respectively, were applied and measured separately within 1 m from the source by a piezoelectric acoustic pressure sensor. The record consists of two heart cycles and two physiological heart tones, S1 and S2, respectively.

To validate the algorithm’s performance, we applied the spectral comparison of both the recorded and the denoised sounds. The analysis is based on the most commonly used Fourier and short-time Fourier transforms along with WT. The spectral analysis was performed on S1 heart tone to determine the particular frequencies in the signal. The short-time transform and wavelet analysis aims to show the particular frequency contents removed by denoising process. The time-frequency representation of the signal shows that the main frequency components of the heart tones remain unchanged. The noise of the lower frequency band (<200 Hz), which overlaps the heart tones spectrum, is removed during the filtration process. It can be seen that the proposed algorithm properly selects the wavelet coefficients associated with the noise. The results show that in the time representation of the recorded signal it is not possible to separate the S1 and S2 components, but after filtration the tones are distinguishable.

## 8. System Verification Based on Automated Diagnostics and QoE

The evaluation/verification process is twofold. First one is a simple classifier, which was used to evaluate the effect of the denoising process on the identification accuracy. It quantitatively evaluates the influence of the developed denoising algorithm on the morphology of the PCG signal. Second one is an online questionnaire, which was designed and distributed amongst stakeholders to quantitatively assess the system’s performance.

The evaluated denoising system WT-TDNN was trained using recordings contaminated by uncorrelated Gaussian white and pink noises, for three noise power levels 5 dBm, 10 dBm, and 15 dBm respectively (see Section 5.1). The classifier used for the evaluation is composed of the Linear Predictive Coding (LPC) and NN [51,52]. The pure original signals from the extended data bases (see Section 5.1) were used in training process of LPC-NN. The test data were contaminated by additive pink or white noises of 5 dBm, 10 dBm, 15 dBm and 20 dBm levels, respectively. After applying the WT-TDNN denoising method, the LPC-NN algorithm was used on the denoised data to identify the given signals origin (e.g., S1, S2, holosystolic murmur, etc.). This way, the robustness of denoising system on noises of different kind and level could be evaluated. The identification correctness is defined as a percentage of correct predictions of considered tone in respect to the total number of samples. The process is repeated for each tone separately. The results from Table 3 show that even strongly contaminated signals after applying the WT-TDNN denoising method could be identified with almost the same accuracy as pure signal, where the reference of identification correctness is this of pure signals. 

Overall, for white noised signals with noise levels of 5 dBm, 10 dBm and 15 dBm, the average identification correctness is 95.7%, 90.4%, and 83.7% for an additive pink noise, respectively; 95.3%, 89.7% and 84.3% for an additive white noise, respectively; compared to 97.3% for a pure origin signal. However, for the pink noise of 15 dBm, the identification correctness varies from 66% for Diastolic Rumble to 90% for S3 and Early Systolic Murmur. Moreover, for white noise of 15 dBm, the identification correctness varies from 69% for Diastolic Rumble to 90% for holosystolic murmur and S4. In these two noise cases, the differences between the best and worst identification correctness are 24% and 21%, respectively, which are 3–7 times worse than for noise levels of 5 dBm and 10 dBm. Overall, the differences in mean value of identification correctness for pink and white noise contaminations are less than 3% for all used noise levels.

An additional test at 20 dBm noise level, which was higher than 15 dBm that used for training was performed. The results show that the average classification correctness for pink and white noise dropped below 65.8% and 67.1%, respectively. For the pink noise, the identification correctness varies from 76% for Early Systolic Murmur and S3 to 45% for Diastolic Rumble. Whereas, for the white noise, the identification correctness changes from 80% for S3 to 45% for Diastolic Rumble. The differences between the best and worst identification correctness are 31% and 36%, respectively. The weak identification capabilities results from the power of the interferences as well as testing and training sets mismatch. The results show that the system performance decreases when the system is trained for lower noise level than the test noise level. However, the results show that the correctness is still at reasonable. Table 4 presents the identification correctness of the LPC-NN classifier applied to signals denoised by the WD white _w_, WD white _p_, WD pink _p_, WD pink _w_ and WT-TDNN algorithms. The results were determined for additive white and pink noises of 10 dBm each. The results of WD white _w_, WD white _p_, and proposed WT-TDNN are similarly good but only for WD algorithms of the same noise distributions as those used in the optimization process. However, the identification correctness obtained for WD algorithms for different noise distributions than those used in the optimization process (WD white _p_ and WD pink _w_) tends to 0%.

Quality of experience (QoE) is a relevant metric for system performance assessment from a user’s perspective. To get a quantitative measure, an online questionnaire was proposed. It was aimed at comparing the performance of the developed algorithm (WT-TDNN) with other popular denoising systems such as the wavelet denoising system optimized for pink noise (WD-pink) [4] and for white (WD-white) [19]. The questionnaire available online [53] was designed in a way that allowed subjective assessment of the sound quality by the listener. The denoising results of these three methods WT-TDNN, WD-white and WD-pink were spread and paired randomly. The played test sounds were results of denoising methods applied to the PCG signal superimposed by 10 dBm noise. The used database was limited to five representative sounds: S3, diastolic rumble (DR), pansystolic murmur (PM), opening snap (OS) and normal split of S2 heard sounds (NS S2).

Table 5 presents the survey averaged results. The detailed results are enclosed in the Appendix B. Each PCG signal resulting from WT TDNN is evaluated in comparison to WD-white and WD-pink. Overall, 36 people took part in the survey, among them 22 engineers, 8 medical students, 3 physicians and 3 people of other specializations.

The results show that 79% of respondents found WT-TDNN as giving a little or much better performance compared to WD-pink, and 83% of them as a little or much better than WD-white. Moreover, 59% of respondents found WT-TDNN as giving much better signal quality than WD-pink and 50% for WD-white. However, 11% and 7% of participants found WD-pink and WD-white, respectively, as giving better performance. A few people could not observe any difference between methods, 3% for WT-pink and 4% for WT-white. The detailed results for each comparison are presented in Appendix B Table A1, Table A2 and Table A3.

## 9. Conclusions and Future Work

The paper objective was to design a denoising algorithm for PCG signals adaptable to changing surrounding interference without compromising its complexity and usability. The proposed solution combining two techniques WT and TDNN, aimed to equally denoise the PCG signal from both white and pink noise, which may affect the heart signal in noisy examination environment. 

The proposed use of TDNN for computation of the IWT achieves comparable results to other wavelet-based denoising systems in terms of SNR and *fit* coefficient. It can be said that the proposed system summarizes both the advantages of blind source separation algorithms (simplicity, reduction of additional sensors) and those of adaptive algorithms (efficiency, accuracy, adaptation to the changes in surrounding environments), and therefore, enables the system implementation on mobile devices.

The best architecture the TDNN consists of 25 neurons in the first and 20 in a second layer with the delay block of 12 samples. The WD parameters, based on previous studies [4,19] are determined as *Coif* 5 wavelet basis at the 10th decomposition level.

The proposed denoising system was modelled and implemented in Matlab. System evaluation and validation were performed on several pathological and physiological heart sounds as well as the signals recorded by mobile devices in a noisy environment and show the usability and diversity of the developed system. The performance of the developed system with respect to other wavelet-based denoising approaches were verified by the online questionnaire.

The proposed system may be used during the development of a portable/wearable cardiovascular monitoring system. Its ability to remove the various PCG noise contaminations may enable the realization of a *smart stethoscope* concept with its implementation on mobile devices like smartphones or tablets. The device miniaturization and portability will allow longer heart examinations without affecting the patient’s quality of life. The longer examination may help to find pathological changes occurring sporadically.

However, the system was only tested on some pathological heart sound signals. Therefore, additional simulations on larger databases including other recordings in various surrounding environments are needed. A further research on a generally trained network can be interesting for system generalisation.

## Figures and Tables

**Table 1:** Details about the dataset used for the simulations and tests.

Base	Michigan		eGeneral		Littmann		Washington		Thinklabs		Sum	
Pathology	CC	*t_r_* [s]	CC	*t_r_* [s]	CC	*t_r_* [s]	CC	*t_r_* [s]	CC	*t_r_* [s]	CC	*t_r_* [s]
S1	156	130	6	4	6	4	10	10	10	10	188	158
S2	156	130	6	4	6	4	10	10	10	10	188	158
S3	167	132	6	4	6	4	11	10	9	8	199	158
S4	160	139	7	5	7	5	12	10	12	10	198	169
EC	155	135	6	4	6	4	-	-	-	-	167	143
NS S1	102	71	6	4	6	4	-	-	-	-	114	79
NS S2	145	132	6	4	6	5	13	10	12	10	182	161
LMS	64	61	7	7	7	7	12	10	16	12	106	97
HM	152	125	7	5	7	7	8	10	11	10	185	157
ESM	63	120	7	5	7	5	-	-	12	10	89	140
OS	75	61	7	5	7	5	-	-	16	11	105	82
DR	75	61	6	4	6	4	11	10	16	11	114	90

CC—a number of cardiac cycles; *t_r_*—record duration.

**Table 2:** Definition of NNs used.

#	Input Layer	First Layer	Second Layer	Output Layer
ANN 1	11	10	-	1
ANN 2	11	15	-	1
ANN 3	11	20	-	1
ANN 4	11	25	-	1
ANN 5	11	25	15	1
ANN 6	11	25	20	1

**Table 3:** The impact of the WT-TDNN denoising on the identification accuracy of LPC-NN classifier.

Type of Heart Dysfunction	Identification Correctness for a Test Signal at Given Noise Power and Colour								
	Pure Signal 0 dBm	5 dBm		10 dBm		15 dBm		20 dBm	
		Pink	White	Pink	White	Pink	White	Pink	White
S1	98%	97%	96%	89%	87%	85%	86%	71%	69%
S2	96%	95%	94%	88%	89%	85%	81%	65%	60%
S3	99%	96%	98%	90%	91%	88%	89%	76%	80%
S4	98%	97%	96%	91%	90%	90%	88%	71%	70%
EC	95%	94%	93%	91%	91%	79%	81%	60%	55%
NS S1	95%	95%	92%	91%	92%	88%	86%	71%	75%
NS S2	96%	95%	96%	90%	87%	79%	79%	55%	50%
LSM	98%	95%	97%	89%	89%	86%	88%	70%	70%
HM	97%	94%	95%	91%	92%	89%	90%	60%	81%
ESM	98%	97%	96%	91%	90%	90%	89%	76%	79%
OS	98%	97%	96%	90%	89%	79%	86%	69%	71%
DR	99%	96%	95%	94%	89%	66%	69%	45%	45%
Mean	97.3%	95.7%	95.3%	90.4%	89.7%	83.7%	84.3%	65.8%	67.1%
Max - Min	4%	3%	6%	6%	5%	24%	21%	31%	36%

where: EC—Ejection Click, NS—Normal Split, LSM—Late Systolic Murmur, HM—Holosystolic Murmur, ESM—Early Systolic Murmur, OS—Opening Snap, DR—Diastolic Rumble.

**Table 4:** Comparison of the identification correctness for different algorithms at white and pink noise of 10 dBm power using following denoising methods: WD white _w_, WD pink _p_, and WT-TDNN.

Type of Heart Dysfunction	Identification Correctness for Different Algorithms at White and Pink Noise of 10 dBm Power					
	WT-TDNN	WT-TDNN	WD White _w_	WD Pink _p_	WD White _w_	WD Pink _p_
	Pink	White	White	Pink	Pink	White
S1	89%	87%	88%	90%	4%	4%
S2	88%	89%	88%	91%	2%	0%
S3	90%	91%	89%	89%	1%	5%
S4	91%	90%	88%	88%	7%	3%
EC	91%	91%	92%	92%	0%	0%
NS S1	91%	92%	90%	92%	1%	2%
NS S2	90%	87%	88%	88%	1%	0%
LSM	89%	89%	87%	87%	0%	0%
HM	91%	92%	87%	87%	0%	0%
ESM	91%	90%	92%	90%	0%	0%
OS	90%	89%	89%	90%	1%	0%
DR	94%	89%	95%	92%	0%	0%
Mean	90.4%	89.7%	89.4%	89.7%	1.4%	1.2%
Max - Min	6%	5%	8%	5%	7%	5%

EC—Ejection Click, NS—Normal Split, LSM—Late Systolic Murmur, HM—Holosystolic Murmur, ESM—Early Systolic Murmur, OS—Opening Snap, DR—Diastolic Rumble.

**Table 5:** Summary of questionnaire with averaging results.

Disclosed Question	WD-Pink	WD-White
The sound treated by WT-TDNN has a little better quality than WD:	20%	33%
The sound treated by WT-TDNN has much better quality than WD:	59%	50%
The sound treated by WT-TDNN has a little bit worse quality than WD:	9%	1%
The sound treated by WT-TDNN has much worse quality than WD:	2%	6%
There is no difference in quality between the signal treated by WT-TDNN and WD:	3%	4%

## Appendix B: Results of the QoE Poll

**Table A1:** Comparison of WT-TDNN with WD-pink.

Question	S3	DR	PM	OS	NS S2
In sound WT-TDNN the heart sound can be heard a little bit better than in sound WD-pink	17%	17%	22%	22%	22%
In sound WT-TDNN the heart sound can be heard much better than in sound WD-pink	72%	56%	39%	67%	61%
In sound WD-pink the heart sound can be heard a little bit better than in sound WT-TDNN	0%	22%	17%	6%	0%
In sound WD-pink the heart sound can be heard much better than in sound WT-TDNN	0%	0%	6%	0%	6%
There is no difference in quality between the signals	0%	11%	6%	0%	0%
In sound WT-TDNN, I can hear some murmurs additive to the heart sound	6%	22%	17%	44%	17%
In sound WD-pink, I can hear some murmurs additive the heart sound	56%	50%	56%	11%	39%

S3—third heart sound; DR—Diastolic Rumble; PM—Pansystolic Murmur; OS—Opening Snap; NS S2—Normal Split of S2.

**Table A2:** Comparison of WT-TDNN with WD-white.

Question	S3	DR	PM	OS	NS S2
In sound WT-TDNN the heart sound can be heard a little bit better than in sound WD-white	28%	50%	28%	22%	39%
In sound WT-TDNN the heart sound can be heard much better than in sound WD-white	61%	28%	61%	50%	50%
In sound WD-white the heart sound can be heard a little bit better than in sound WT-TDNN	0%	6%	0%	0%	0%
In sound WD-white the heart sound can be heard much better than in sound WT-TDNN	0%	0%	0%	17%	11%
There is no difference in quality between the signals	0%	11%	6%	6%	0%
In sound WT-TDNN, I can hear some murmurs additive to the heart sound	28%	44%	22%	22%	11%
In sound WD-white, I can hear some murmurs additive the heart sound	56%	28%	44%	44%	39%

S3—third heart sound; DR—Diastolic Rumble; PM—Pansystolic Murmur;OS—Opening Snap; NS S2—Normal Split of S2.

**Table A3:** Comparison of WD-pink with WD-white.

Question	S3	DR	PM	OS	NS S2
In sound WD-pink the heart sound can be heard a little bit better than in sound WD-white	17%	33%	39%	50%	44%
In sound WD-pink the heart sound can be heard much better than in sound WD-white	11%	17%	0%	17%	33%
In sound WD-white the heart sound can be heard a little bit better than in sound WD-pink	28%	11%	11%	11%	17%
In sound WD-white the heart sound can be heard much better than in sound WD-pink	11%	0%	6%	0%	11%
There is no difference in quality between the signals	11%	28%	28%	50%	28%
In sound WD-pink, I can hear some murmurs additive to the heart sound	11%	17%	17%	6%	0%
In sound WD-white, I can hear some murmurs additive the heart sound	39%	39%	33%	11%	0%

S3—third heart sound; DR—Diastolic Rumble; PM—Pansystolic Murmur; OS—Opening Snap; NS S2—Normal Split of S2.
